# Risk Assessment and Management of *Brucella canis* Introduction via Commercial Dog Imports Into France

**DOI:** 10.1111/risa.70217

**Published:** 2026-03-14

**Authors:** Patrick Mvumbi, Gina Zanella, Guillaume Crozet

**Affiliations:** ^1^ Anses, École nationale vétérinaire d'Alfort, Laboratoire de Santé Animale EPIMIM Maisons‐Alfort France; ^2^ Université Paris‐Est Créteil (UPEC) Créteil France

**Keywords:** *Brucella canis*, disease introduction, dogs, management measures, quantitative risk assessment

## Abstract

Canine brucellosis, caused by *Brucella canis*, is a likely underdiagnosed zoonotic disease that leads to reproductive failure in dogs and economic losses for kennels. Since 2020, cases in mainland France have been on the rise, with most attributed to the importation of dogs from Eastern Europe. However, the risk of introducing *B. canis* into mainland France through commercial dog imports from worldwide sources remains poorly characterized. To address this gap, a quantitative risk assessment was conducted using a stochastic scenario tree model incorporating the region of origin of the imported dogs. In addition, impact of management measures on the annual number of *B. canis*‐infected dogs imported into France was evaluated. The results showed that the commercial dog imports represent a substantial risk for introducing *B. canis* into mainland France, with a median annual number of infected dogs imported estimated at 41.60 (95% PI: 5.12–8784.00). This risk was particularly high for imports originating from the USA, Eastern Europe, North Africa, and Latin America. The evaluation of management measures through simulations showed that the introduction of mandatory pre‐import testing for *B. canis*, with 80% compliance or full enforcement—could significantly reduce the associated risk. These measures led to a reduction in the annual number of infected dogs imported into mainland France by a factor of 4 to 1000 compared to the baseline model. These findings highlight the need for implementing targeted management measures to prevent the introduction of *B. canis* into France.

## 1 Introduction

Canine brucellosis is a likely underdiagnosed zoonotic disease caused by *Brucella canis*. Humans can be infected with *B. canis*, which typically causes mild, nonspecific symptoms, although severe cases have also been reported (Weese and Weese 2025; Ying et al. 1999). Individuals at higher risk of exposure include those who handle breeding dogs in kennels or are exposed to their reproductive tissues and fluids, such as veterinarians and laboratory personnel (Krueger et al. 2014; Lucero, Escobar, et al. 2005; Lucero, Jacob, et al. 2005; Marzetti et al. 2013; Süer et al. 2023).

In dogs, transmission can occur through the oral, nasal or conjunctival mucosae, via sexual contact (Brower et al. 2007; Carmichael and Joubert 1988; Olsen and Palmer 2014; Santos et al. 2021; Wanke 2004), or vertically intrauterine or through lactation (Makloski 2011; Santos et al. 2021; Wanke 2004). It is a major animal health concern, particularly in canine breeding populations, due to the resulting severe reproductive problems. Infected female dogs are prone to developing metritis, placentitis, and late‐term abortions, while males often suffer from epididymitis, orchitis, and prostatitis (Carmichael 1976; Carmichael and Joubert 1988; Djokic et al. 2023; Hollett 2006; Santos et al. 2021; Wanke 2004). In addition to causing abortions, *B. canis* can lead to infertility in both sexes, resulting in significant economic losses for breeding kennels, where reproductive success is essential (Makloski 2011). Other commonly reported clinical manifestations of *B. canis* infection in dogs include discospondylitis (Anderson and Binnington 1983; Corrente et al. 2010; Djokic et al. 2023; Forbes et al. 2019; Kerwin et al. 1992; Long et al. 2022), generalized lymphadenopathy and uveitis (Djokic et al. 2023; Holst et al. 2012; Wanke 2004).

Several methods are available to diagnose *B. canis* infection in dogs, including serology, blood and genital bacterial cultures, and polymerase chain reaction (PCR). *B. canis* serological tests and PCR are widely available to veterinarians through commercial diagnostic laboratories. However, diagnosis is challenging due to the nonspecific nature of clinical signs, the high prevalence of asymptomatic infections, and the limited reliability and accuracy of currently available laboratory diagnostic methods (Baldi et al. 1994; Carmichael and Shin 1996; Cosford 2018; Djokic et al. 2023; Keid 2007, 2009; Keid et al. 2015, 2017; Mateu‐de‐Antonio and Martín 1995; Mateu‐de‐Antonio et al. 1993, 1994; Mol et al. 2020; Perletta et al. 2023; Wanke 2004; Wanke et al. 2002; Zoha and Carmichael 1982).

The burden of this infection on canine breeding industry is exacerbated by the unavailability of a vaccine and the lack of effective treatment options. Treatment of infection requires prolonged administration of multiple antimicrobials over several months. However, there is a high risk of relapse following only a brief remission (Djokic et al. 2023; Hollett 2006; Santos et al. 2021). Moreover, infected dogs remain a potential source of infection for other dogs and for humans for several years (Hollett 2006; Santos et al. 2021; Wanke 2004). Preventing the introduction of *B. canis* into breeding facilities is therefore essential to avoid the significant economic costs associated with managing this infection. The combined impact on canine and human health and kennel economics highlights the need for monitoring and control measures, especially in breeding environments.

Canine brucellosis is well established as an enzootic disease in certain regions worldwide, including the southern United States, Central and South America (Bezerra et al. 2012; Boeri et al. 2008; Colman et al. 2017; De Paula Dreer et al. 2013; Dorneles et al. 2011; A. Fernandes et al. 2013; A. R. F. Fernandes et al. 2011; Laverde et al. 2021; López et al. 2009; Maza V. and Morales C. 2016; Ruiz et al. 2010; Santana et al. 2013; Schiavo et al. 2024; Suárez‐Esquivel et al. 2021; Troncoso et al. 2013; Vasconcelos et al. 2008), Asia (Alshehabat et al. 2019; Jiang et al. 2012; Kume et al. 2013; Mathur et al. 2008; Mosallanejad et al. 2009; Öncel et al. 2005; Özavci et al. 2023; Parin et al. 2020; Xiang et al. 2013; Yoak et al. 2014), Africa (Anyaoha et al. 2020; Cadmus et al. 2011; Chinyoka et al. 2014; Hamdy et al. 2023; Oosthuizen et al. 2019), and Eastern Europa (Buhmann et al. 2019; Cosford 2018; Hensel et al. 2018; Santos et al. 2021; Wanke 2004). However, the true prevalence of this infection in these regions remains poorly characterized. Recently, it has emerged as a growing threat in Western European countries, including France, where positive dogs have been detected since 2020 in ten breeding kennels across various regions. Most of these cases have been linked to online purchases of dogs from Eastern European countries. In 2022, the High Council of Public Health (“Haut Conseil de la Santé Publique—HCSP”) conducted the first risk assessment in France, focusing on the risk for humans exposed to infected dogs. This assessment followed several reported cases in dogs imported from Eastern Europe (Russia, Belarus, and Romania) and the USA since December 2021 (Haut Conseil de la Santé Publique 2022). However, it focused exclusively on the risk to humans, using a qualitative approach, and did not consider the exposure risk to dogs from the importation of infected animals. Therefore, the primary objective of this study was to quantitatively assess the risk of introducing *B. canis* into mainland France through commercial dog imports.

This is particularly important given the regulatory context. Unlike other *Brucella* species, *B. canis* is not a notifiable disease under the Animal Health Law in European Union (EU) countries. Although it is notifiable in France, no specific control measures have been implemented. Furthermore, despite reports of *B. canis* in imported dogs, there are still no requirements for pre‐import screening in dogs even from areas where canine brucellosis is considered enzootic. In response, this study also aimed to evaluate potential management measures to reduce the risk of its introduction.

## 2 Materials and Methods

A quantitative risk assessment (QRA) was conducted using a stochastic scenario tree model (Vose 2010) that explicitly incorporated the region of origin of the imported dog (and its level of *B. canis* infections prevalences).

### 2.1 Country Grouping

To gain insights into the risk associated with different regions of the world involved in the commercial dog trade to France, countries were grouped into 12 clusters. This process allowed to quantify the risk associated with each cluster of countries. The clusters were defined based on geographical proximity, the epidemiology of canine brucellosis, and the organization of veterinary services. Each cluster included only countries from which France imported dogs for commercial purposes between 2021 and 2023, as recorded in the French domestic carnivore database (I‐CAD). This database records the importation of domestic carnivores into France, and distinguishes between importations by professionals and private individuals. For this study, we included dogs imported by professionals, since this category most closely reflects commercial dog imports and serves as the best available proxy for breeding dogs. Although it may include some animals not intended for reproduction, it excludes pets brought in by private individuals for companionship, adoption or resale. This subset is therefore the most relevant for assessing the risk of introduction through professional breeding channels. Table 1 shows the 12 clusters of countries included in the QRA.

**TABLE 1:** Country clusters considered in the quantitative risk assessment.

Cluster of countries	Countries
Western Europe	Austria, Belgium, Germany, Luxembourg, Monaco, the Netherlands, Switzerland
Eastern Europe	Albania, Belarus, Bulgaria, Czech Republic, Croatia, Hungary, Moldavia, Poland, Romania, Russia, Slovakia, Slovenia, Ukraine
Northern Europe	Denmark, Estonia, Finland, Iceland, Ireland, Latvia, Lithuania, Norway, United Kingdom, Sweden
Mediterranean region of Europe	Andorra, Vatican City, Greece, Gibraltar, Italy, Malta, Portugal, Spain
Western Asia	Afghanistan, Armenia, Cyprus, UAE, Israel, Iraq, Iran, Lebanon, Oman, Pakistan, Qatar, Syria, Turkey
East Asia 1	Cambodia, China, India, Indonesia, Malaysia, Thailand, Vietnam
East Asia 2	Japan, Hong Kong, Republic of Korea, Taiwan
Sub‐Saharan Africa	Cameroon, Cape Verde, Gabon, Kenya, Madagascar, Mauritius, Nigeria, Senegal, South Africa
North Africa	Algeria, Egypt, Morocco, Mauritania, Tunisia
Latin America	Argentina, Bolivia, Brazil, Chile, Ecuador, Mexico, Peru, Puerto Rico, Venezuela
USA	United States of America
Canada	Canada

### 2.2 Risk Pathways

**FIGURE 1:**
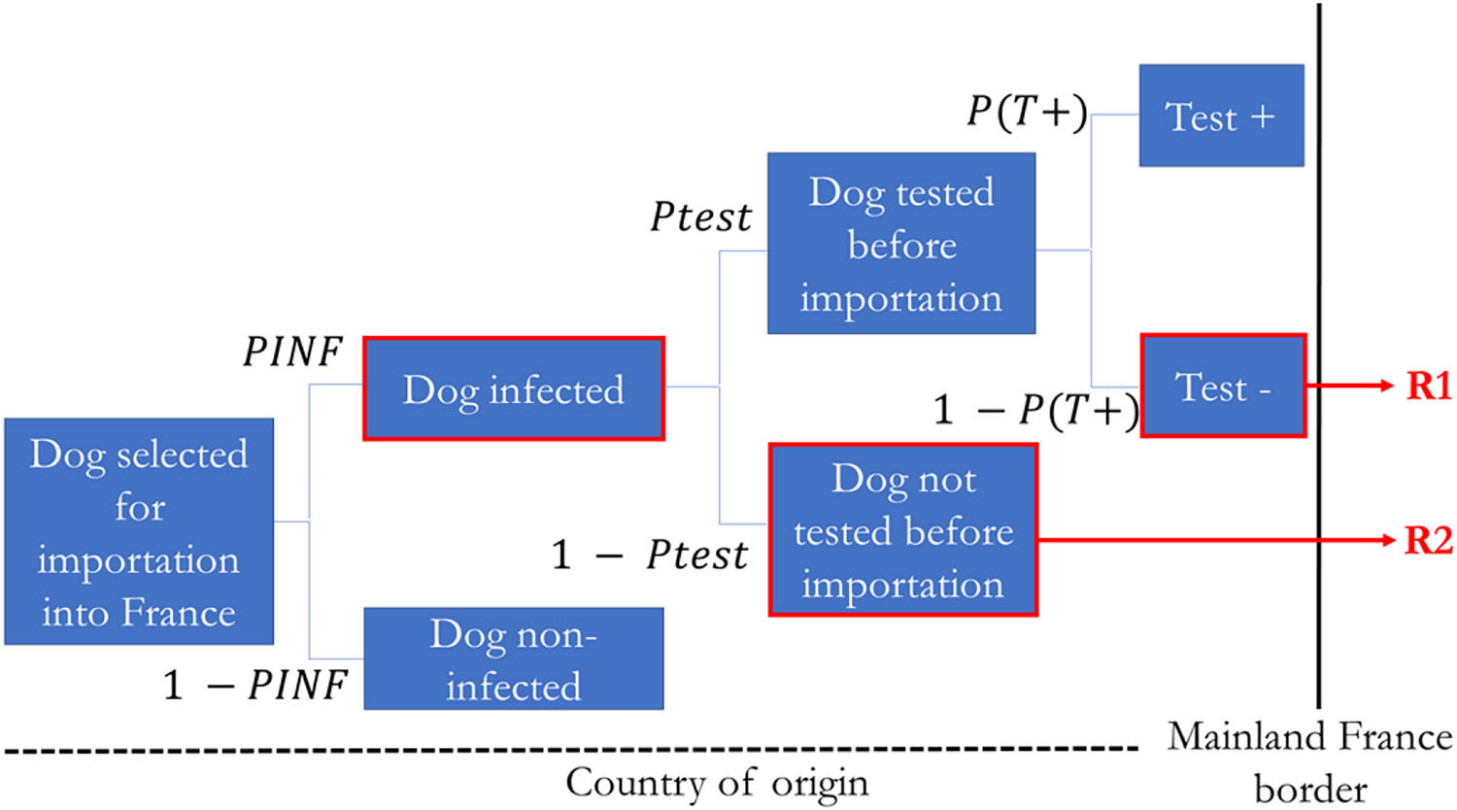
Risk pathways (scenario tree) for the entry of *Brucella canis* into mainland France following the commercial dog imports where, *PINF* = probability that a dog is infected, *Ptest* = probability of testing a dog selected for import and *P(T+)* = probability of a positive test outcome in an infected dog. The boxes outlined in thick red represent the adverse events leading to the risk. *R1* (in red) represents a scenario in which a dog is tested but not identified as infected due to the test's limited sensitivity, allowing its entry into mainland France. *R2* (in red) depicts the introduction of an infected dog into mainland France without any testing.

A schematic diagram of the model structure was constructed, detailing the sequential events necessary for the entry of *B. canis*. The scenario tree model presented in Figure 1 outlines two potential pathways for the introduction of *B. canis* into mainland France, starting with the selection of a dog in the country of origin. It details the sequence of events that could lead to the importation of an infected dog.

In this baseline scenario, we assume that if a dog tests positive during pre‐import testing, it is excluded from the import process and does not enter France. This reflects an idealized situation in which pre‐import testing acts as an effective barrier. Consequently, the model structure does not include the potential import pathway despite a positive test result.

### 2.3 Parameterization of the Model

#### 2.3.1 Probability of Infection

The true seroprevalence of *B. canis* infection (i.e., seroprevalence adjusted for sensitivity and specificity of the diagnostic test used) in each country cluster was used as an estimate of the probability of infection, assuming it remained stable and accurately reflected the current epidemiological status. This seroprevalence was estimated using data gathered from two sources: literature on *B. canis* apparent seroprevalence published since 2000 (Appendix SA1) and test results from a European veterinary diagnostic laboratory for *B. canis* (Buhmann et al. 2019) (Appendix SA2).

With regard to published surveys, we excluded studies based on outbreak investigations or targeting high‐risk populations (e.g., contact tracing or clinical cases), as these are likely to overestimate the true prevalence. We retained only those studies that reported seroprevalence in broader or more defined populations (e.g., shelter dogs or routine diagnostic submissions), while carefully considering the study context (Appendix SA1).

In parallel, test results were extracted from the database of a veterinary diagnostic laboratory (IDEXX Laboratories, Ludwigsburg, Germany), which received *B. canis* test samples from dogs across 20 European countries between 2011 and 2016. Buhmann et al. (2019) previously analyzed and published these data, which were used in this QRA to estimate posterior estimates of seroprevalence in clusters of European countries due to the lack of directly comparable seroprevalence surveys in the region (Appendix SA2). The country of origin of each sample is explicitly reported in the study by Buhmann et al. (2019). In our QRA, we relied strictly on the reported country of origin to assign seroprevalence values to country clusters. Due to the limited epidemiological data available for *B. canis* in Europe, this dataset is one of the few sources that can inform regional estimates.

To address the imperfections of diagnostic tests for *B. canis* used in seroprevalence surveys, Bayesian latent class analysis (BLCA) was used to estimate the true seroprevalence for each country cluster (Branscum et al. 2005; Cheung et al. 2021; Kruschke 2015). A BLCA was performed for each formal seroprevalence survey. When multiple surveys were available within a country cluster, the results were combined (e.g., Western Asia: six surveys; East Asia 1: four surveys; East Asia 2: two surveys; Latin America: 28 surveys; USA: three surveys; Canada: one survey; sub‐Saharan Africa: five surveys; North Africa: one survey) (Appendix SA1). For European clusters where no formal surveys were available, data points derived from targeted screening contexts, such as testing imported dogs or clinical suspects, were used to estimate seroprevalence (e.g., Northern Europe: four data points; Western Europe: five data points; Eastern Europe: two data points; Mediterranean Europe: one data point) (Appendix SA2).

In this approach, a dog's true infection status was the latent variable; in other words, it was considered unobserved. The posterior distribution of the probability of *B. canis* infection was estimated through a combination of observed diagnostic test outcomes (observed data), prior information on the sensitivity and specificity of the serological test(s) and noninformative prior information on the disease seroprevalence by country cluster. Practically, the following steps were followed:

i. Specification of prior distributions for true seroprevalence, test sensitivity, and test specificity

We specified the prior distribution for true seroprevalence as a noninformative uniform beta distribution (Beta(1, 1)), which implies that all seroprevalence values between 0 and 1 were considered equally likely a priori. We applied uninformative priors deliberately to avoid biasing the posterior estimates toward nonrepresentative prior assumptions. This approach enabled the posterior distributions to more accurately reflect the inherent uncertainty of the study designs and population characteristics of the data sources. Conversely, informative beta prior distributions were used to account for the uncertainty in the sensitivity and specificity of the different *B. canis* serological tests. Published data on the sensitivity and specificity of these tests, including their minimum and maximum values (Appendices SB and SC), were used to estimate the parameters for their prior beta distributions. The fifth percentile values for these estimates were derived from findings in the relevant literature. The parameters for these beta prior distributions were then calculated using this information and the Epi Beta Buster application (available at https://shiny.vet.unimelb.edu.au/epi/beta.buster/).

ii. Specification of the likelihood function for the observed data

The methods described by Branscum et al. (2005) were used to define the likelihood functions for the observed data. In the studies selected, either a single test or two tests were used to estimate the apparent seroprevalence within a single population. For the one‐test, single population case, the likelihood function *y*_1_ was defined in Equation (1) as a binomial distribution:

(1) $$y_{1}∼\mathrm{binomial}\left(n,\;p\right)\;$$

where *n* is the number of dogs randomly sampled from a population and *p* the probability of a dog testing positive (P(T+)) defined in Equation (2) as:

(2) $$p=P\;\left(T+\right)=\left(\pi \times Se\right)+\left(\left(1-\;\pi \right)\times \left(1-Sp\right)\right)$$

with π, the true seroprevalence, *Se*, the sensitivity, and *Sp* the specificity of the test.

For the two‐test, single population case, a Bayesian conditional‐dependence model was implemented which accounts for the covariances between the two tests (Dendukuri and Joseph 2001), as the tests measure the same biological process: the presence of anti‐*B. canis* antibodies. The likelihood function *y*_2_ defined in Equation (3), follows a multinomial distribution:

(3) $$y_{2}∼\mathrm{multinomial}\;\left(n,\;\left(p11,\;p12,\;p21,\;p22\right)\right)\;$$

where *p*_11_ is the probability that the results for both tests (*T*_1_ and *T*_2_) are positive, *p*_12_ the probability that *T*_1_'s result is positive and *T*_2_'s is negative; *p*_21_ the probability that *T*_1_'s result is negative and *T*_2_'s positive and *p*_22,_ the probability that both test results are negative.

iii. Calculation of posterior estimates for true seroprevalence, test sensitivity and test specificity

A Bayesian analysis was conducted to estimate the posterior distributions of the true seroprevalence surveys, tests sensitivity and test specificity. For country clusters with multiple posterior distributions (i.e., for which multiple studies providing apparent seroprevalences, see Section 2.3.1), these were combined using a discrete distribution that assigned equal weight to each posterior distribution.

#### 2.3.2 Probability of Testing a Dog Selected for Import

The probability of testing a dog selected for import (Figure 1) was set at 0.01. This conservative estimate reflects the minimum probability, as testing for *B. canis* is not currently required before import.

#### 2.3.3 Probability of a Positive Test Outcome

Two serological tests, the microscopic agglutination test (MAT) and lateral flow immunochromatographic assay (LFIA) were assumed to be used to screen dogs before import, following the protocol of the French National Reference Laboratory for Brucellosis. These tests were interpreted as parallel tests, meaning a dog was considered *B. canis* seropositive if at least one test was positive. The posterior distributions of test sensitivity for these tests derived from the BLCA (Table 2) were incorporated into the model to estimate the probability of a positive screening outcome.

**TABLE 2:** Posterior estimates of micro agglutination test (MAT) and lateral flow immunochromatographic (LFIA) sensitivity and specificity from Bayesian latent class analysis.

	Median posterior sensitivity (95% probability interval)	Median posterior specificity (95% probability interval)
MAT	0.53 (0.40–0.65)	0.97 (0.94–0.99)
LFIA	0.92 (0.73–0.99)	0.97 (0.95–1.00)

#### 2.3.4 Average Annual Number of Dogs Imported

We used a deterministic value calculated as the mean over 3 years (2021–2023) of the number of dogs imported for commercial purposes to estimate the average annual number of imported dogs, using data from the national I‐CAD database (Appendix SD). These animals represent the population most relevant to the risk of introducing *B. canis* into breeding settings. However, not all imported dogs are registered, and some may be misclassified. Therefore, these figures likely underestimate the actual number of commercial imports. Our risk assessment is based on this conservative assumption and represents a lower‐bound estimate of the risk associated with the international dog trade.

### 2.4 Risk Assessment (Baseline Model)

Based on the defined scenario tree (Figure 1), the probability of occurrence of the risk pathways *R1* and *R2* was calculated, respectively, as in Equations (4) and (5), for each country cluster (*c*) by considering the probabilities of the events within both pathways:

(4) $$R1_{c}=\mathrm{PIN}F_{c}\times \mathrm{Ptest}\times \left(1-P\left(T+\right)\right)$$

and

(5) $$R2_{c}=PINF_{c}\times \left(1-Ptest\right)$$

where *PINF_C_* represents the probability that a dog is infected in country cluster (*c*)*; Ptest* is the probability of testing a dog selected for import and *P(T+)* denotes the probability of a positive test outcome for an infected dog.

The probability that a dog imported from a country cluster (*c*) is infected (*RIMP_C_*) was calculated as in Equation (6):

(6) $$\mathrm{RIM}P_{c}=R1_{c}+R2_{c}\;$$

The annual number of infected dogs imported into mainland France was then calculated by country cluster (*NINF_C_*) as in Equation (7):

(7) $$\mathrm{NIN}F_{c}=\mathrm{RIM}P_{c}\times \mathrm{NIM}P_{c}\;$$

where *RIMP_C_* is the probability that a dog imported from a country cluster (*c*) is infected and *NIMP_C_* represents the average number of dogs imported annually into mainland France for commercial purposes from a country cluster *(c*). Finally, the global risk, or the annual number of infected dogs imported into mainland France from across the world (*NINF*), is described in Equation (8):

(8) $$NINF=\sum_{c=1}^{12}NINF_{c}\;$$

where *NINF_C_* is the annual number of infected dogs imported into mainland France from country cluster (*c*).

### 2.5 Sensitivity Analysis

A sensitivity analysis using Spearman's correlation, a rank‐based correlation measure, was conducted to identify the input parameters whose uncertainty or variability has the greatest impact on the uncertainty or variability of the output parameter *NINF*.

### 2.6 Alternative Scenarios

#### 2.6.1 Description of the Management Measures

The impact of two management measures on the annual number of *B. canis*‐infected dogs imported into France was assessed by modifying the structure of the baseline QRA model and adjusting specific parameters.

**FIGURE 2:**
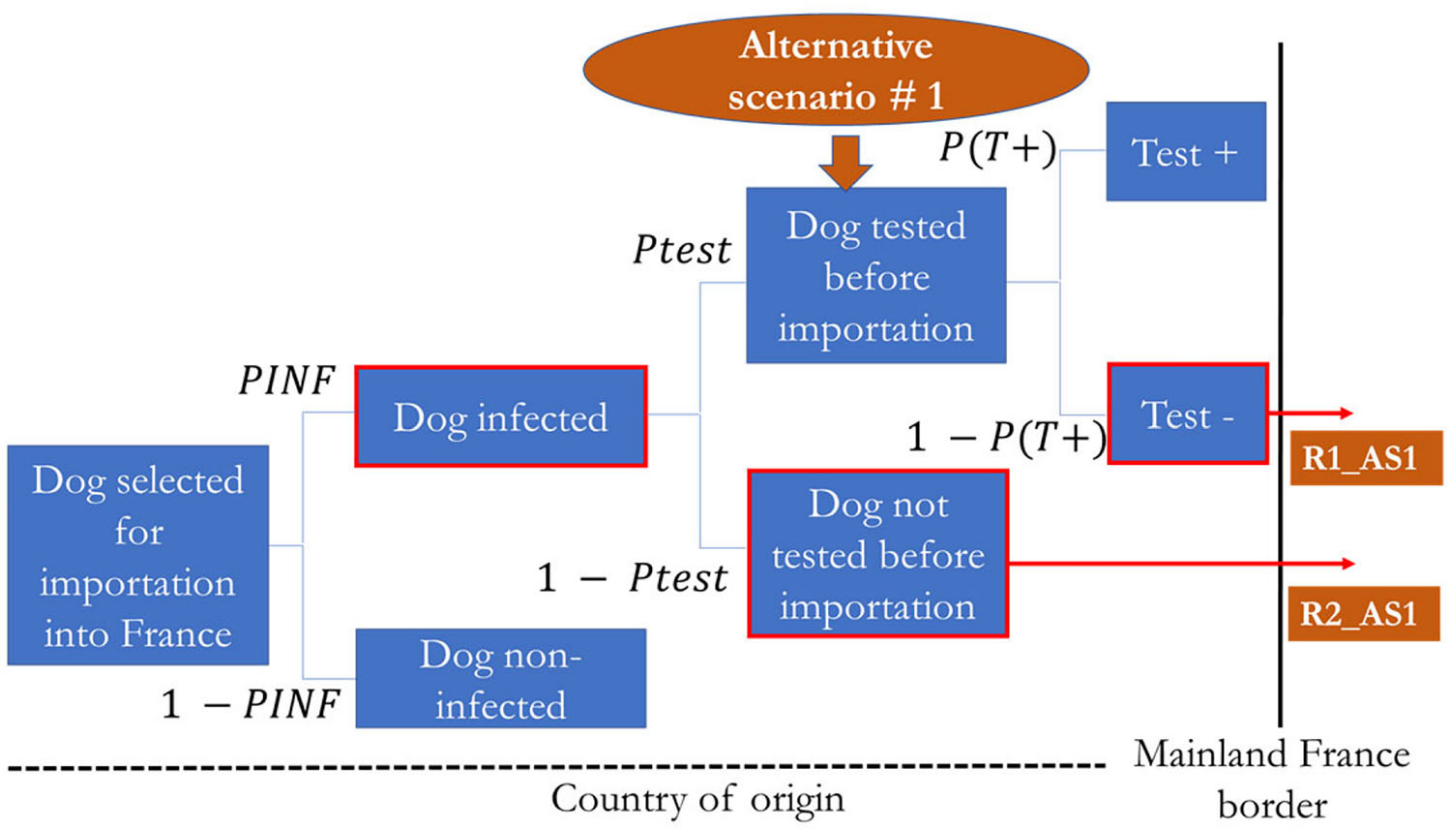
Alternative Scenario #1 evaluated—mandatory pre‐import testing for *Brucella canis* where *PINF* = probability that a dog is infected, *Ptes*t = probability of testing a dog selected for import and *P(T+)* = probability of a positive test outcome in an infected dog. The boxes outlined in thick red represent the adverse events leading to the risk. The brown circle represents the preventive measure. The two outcomes of alternative Scenario #1 are shown separately. *R1_AS1* and *R2_AS1* appear in separate brown boxes and correspond to the first and second outcomes of alternative Scenario #1, respectively.

**FIGURE 3:**
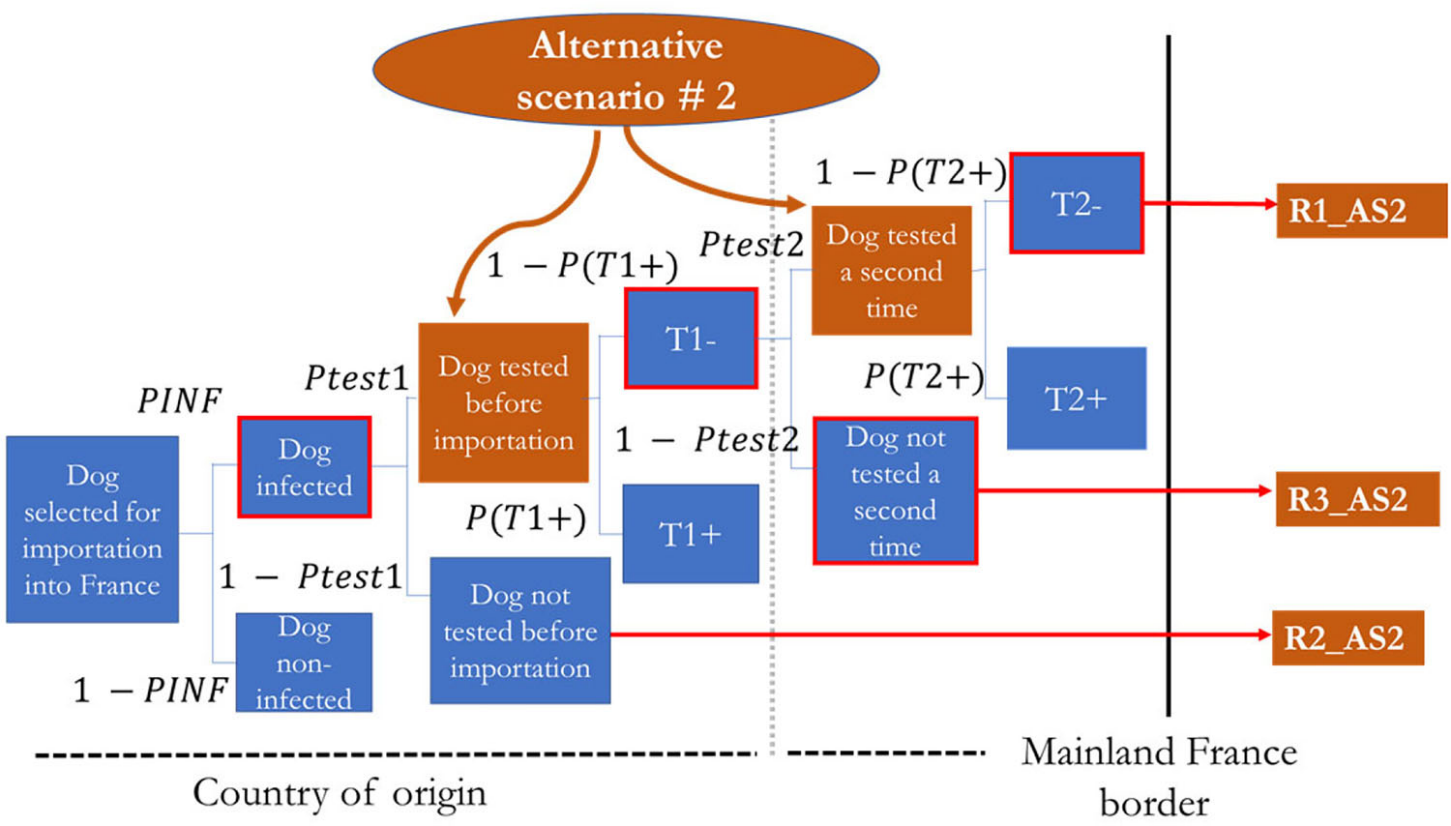
Alternative Scenario #2 evaluated—Combination of mandatory pre‐import testing and additional testing at the French border. The brown circle represents the preventive measures. This scenario combines mandatory pre‐import testing with an additional test upon arrival at the French border. Where *PINF* = probability that a dog is infected; *Ptest1* = probability of testing a dog before import; *Ptest2* = probability of testing a dog at the French border; *P(T1^+^)* = probability that an infected dog tests positive on the pre‐import test; *P(T2^+^)* = probability that an infected dog tests positive on the second test.

We assumed a mandatory pre‐import testing policy involving either a single test or a two‐step testing protocol, depending on the scenario considered. However, recognizing that full enforcement may not always be achievable under real‐world conditions, three levels of compliance with this requirement were modeled under alternative Scenario #1: 50%, 80%, and 100%; and two levels of compliance under alternative Scenario #2: 80% and 100%. These values reflect potential variability in adherence to the regulation, which may arise from factors such as limited oversight, undeclared imports, or logistical constraints. This modeling approach enables us to evaluate the policy's effectiveness under ideal and suboptimal implementation scenarios. The two management measures evaluated are illustrated in Figures 2 and 3 and are as follows:

1. Mandatory pre‐import testing for *B. canis* with compliance levels set at 50%, 80%, and 100%, and;
2. Mandatory additional testing at the French border with compliance levels of 80% and 100%. The second test was assumed to be conducted 2–4 weeks after the initial screening and was intended to detect seroconversion or identify any technical or biological errors from the first test. Dogs were assumed to be held temporarily under quarantine conditions until the results of the second test became available to prevent potential transmission during the diagnostic window. This temporary retention was implicitly included as part of the testing procedure.

Accordingly, we simulated two alternative scenarios:

Scenario 1 : : Mandatory pre‐import testing for *B. canis* only (Figure 2).
Scenario 2 : : Involved a combination of mandatory pre‐import testing and additional testing at the French border (Figure 3). This approach was based on post‐entry quarantine measures similar to that implemented in Australia.

These two scenarios were designed to evaluate how different combinations of control measures could reduce the risk of importing *B. canis*‐infected dogs to mainland France.

As shown in Figure 2, alternative Scenario #1 results in two outcomes:

The first outcome, *R1_AS1*, involves mandatory pre‐import testing for *B. canis* with three levels of compliance:

With 50% compliance, testing is performed in the exporting country in one out of two cases. However, infected dogs may still enter mainland France due to incomplete implementation of the testing requirement and limited test sensitivity.
With 80% compliance, testing is performed in four out of five cases. Similarly, infected dogs may enter mainland France for the same reasons.
With 100% compliance, testing is performed in all cases in the exporting country. In this case, the entry of infected dogs into mainland France is solely due to the tests' limited sensitivity.

*R2_AS1* represents the second outcome, in which a dog is not tested despite the mandatory requirement, thereby allowing entry into mainland France.

As shown in Figure 3, alternative Scenario #2 has three possible outcomes.

*R1_AS2* is the first outcome and involves mandatory pre‐import testing combined with additional testing at the French border. Each preventive measure is applied at two levels of compliance: 80% and 100%.

— At 80% compliance, both measures are applied in four out of five cases. However, infected dogs may still enter mainland France due to the incomplete application of the measures and the limited sensitivity of the tests.
— At 100% compliance, both measures are applied without exception. In this case, the entry of infected dogs is solely due to the tests' limited sensitivity.

*R2_AS2* corresponds to the second outcome, in which no testing is performed despite the mandatory requirement, thereby allowing infected dogs to enter mainland France.

*R3_AS2* represents the third outcome, involving only mandatory pre‐import testing (i.e., no additional testing at the French border). There are two levels of compliance:

— With 80% compliance, the test is applied in four out of five cases. Infected dogs may still enter due to incomplete implementation and limitations in test sensitivity.
— Even at 100% compliance, when the measure is fully applied, infected dogs may still enter due to the limited sensitivity of the test.

Alternative Scenario #2 adds a second diagnostic opportunity to highlight the added value of repeated testing in mitigating the limitations of single‐time‐point serological screening.

#### 2.6.2 Risk Assessment After Implementation of the Alternative Scenario

##### 2.6.2.1 Alternative Scenario #1

Based on the defined scenario tree for alternative Scenario #1 (Figure 2), the probability of occurrence of the risk pathways *R1_AS1* and *R2_AS1* was calculated following the same approach as in the baseline model (Section 2.4). The same equations apply, except that *Ptest* was replaced by *Ptest_i_*, which represents the probability of testing a dog selected for importation under a specific compliance level (*i*). For clarity, three levels of compliance with mandatory pre‐import testing were modeled under alternative Scenario #1, compared to the 1% compliance used in the baseline scenario: 50%, 80%, and 100%. Two levels of compliance were considered under alternative Scenario #2: 80% and 100%.

##### 2.6.2.2 Alternative Scenario #2

Based on the defined scenario tree for alternative Scenario #2 (Figure 3), the probabilities of occurrence of the risk pathways *R1_AS2*, *R2_AS2*, and *R3_AS2* were calculated respectively as in Equation (9)–(11), for each country cluster (*c*) under a specific compliance level (*i*), by considering the probabilities of the events within both pathways:

(9) $$R\;1\_AS2_{c,i}=PINF_{c}\times Ptest1i\times \left(1-P\left(T1+\right)\right)\times Ptest2i\times \left(1-P\left(T2+\right)\right)$$

and

(10) $$R2\_AS2_{c,i}=PINF_{c}\times \left(1-Ptest1i\right)$$

and

(11) $$R\;3\_AS2_{c,i}=PINF_{c}\times Ptest1i\times \left(1-P\left(T1+\right)\right)\times \left(1-Ptest2i\right)$$

where *PINF_C_* represents the probability that a dog is infected in country cluster (*c*)*; Ptest1, i*: probability of testing a dog before import based on compliance level (*i*), *Ptest2, i*: probability of testing a dog at the French border based on compliance level (*i*), *P(T1+)*: probability of an infected dog testing positive on the pre‐import test, *P(T2+)*: probability of an infected dog testing positive on the additional test at the French border.

The probability that a dog imported from a country cluster (*c*) based on compliance level (*i*) is infected after the implementation of the alternative Scenario #2 (*RIMP_AS2c, i*) was calculated as in Equation (12):

(12) $$\mathrm{RIMP}\_\mathrm{AS}2_{c,\;i}=R1\_\mathrm{AS}2_{c,i}+R2\_\mathrm{AS}2_{c,i}+R3\_\mathrm{AS}2_{c,i}$$

The annual number of infected dogs imported into mainland France under a specific compliance level (*i*) after the implementation of the alternative Scenario #2 was then calculated by country cluster (*NINF_AS2c, i*) as in Equation (13):

(13) $$\mathrm{NINF}\_\mathrm{AS}2_{c,\;i}=\mathrm{RIMP}\_\mathrm{AS}2_{c,i}\times \mathrm{NIMc}$$

where *RIMP_AS2c, i* is the probability that a dog imported from a country cluster (*c*) under a specific compliance level (*i*) is infected after the implementation of the alternative Scenario #2 and *NIMP_C_* represents the average number of dogs imported annually into mainland France for commercial purposes from a country cluster (*c*). Finally, the global risk, or the annual number of infected dogs imported into mainland France from across the world under a specific compliance level (*i*) *(NINF_AS2i*) after the implementation of the alternative Scenario #2, is described in Equation (14):

(14) $$NINF\_AS2i=\sum_{c=1}^{12}NINF\_AS2_{c,i}$$

where *NINF_AS2c, i* is the annual number of infected dogs imported into mainland France from country cluster (c) under a specific compliance level (*i*) after the implementation of the alternative Scenario #2.

##### 2.6.2.3 Estimation of Outcome and Impact Metrics

In addition to estimating the number of *B. canis*‐infected dogs introduced under each scenario, we computed two complementary impact indicators: the risk ratio (RR) and the absolute risk reduction.

The RR was used to compare the relative risk of introduction under each intervention scenario compared to the baseline (i.e., no mitigation). It was calculated as follows for each alternative scenario and compliance level (*i*):

The calculation is detailed in Equation (15) for alternative Scenario #1 under a given compliance level (*i*), and in Equation (16) for alternative Scenario #2 under the same conditions:

(15) $${\mathrm{RR}}_{\mathrm{AS}1i}=\frac{\mathrm{NINF}\_\mathrm{AS}1_{i}}{\mathrm{NINF}}$$

(16) $${\mathrm{RR}}_{\mathrm{AS}2i}=\frac{\mathrm{NINF}\_\mathrm{AS}2_{i}}{\mathrm{NINF}}$$

where *NINF* represents the annual number of infected dogs imported into mainland France under the baseline scenario; *NINF_AS1i* and *NINF_AS2i* represent the annual number of infected dogs imported into mainland France under alternative Scenarios #1 and #2, respectively, and under a specific compliance level (*i*).

To provide a more tangible measure of impact, we also calculated the absolute risk reduction for 1000 dogs imported (*ARR*) under each intervention scenario. *ARR* was defined as the difference in the number of *B. canis*‐infected dogs per 1000 imported dogs between the baseline and the alternative scenario.

The calculation is detailed in Equation (17) for alternative Scenario #1 under a given compliance level (*i*), and in Equation (18) for alternative Scenario #2 under the same conditions:

(17) $$\mathrm{AR}R_{\mathrm{AS}1i}=\left[\frac{\mathrm{NINF}-\mathrm{NINF}\_\mathrm{AS}1_{i}}{\mathrm{NIMP}}\right]\times 1000$$

(18) $$\mathrm{AR}R_{\mathrm{AS}2i}=\left[\frac{\mathrm{NINF}-\mathrm{NINF}\_\mathrm{AS}2_{i}}{\mathrm{NIMP}}\right]\times 1000$$

where *NINF* represents the annual number of infected dogs imported into mainland France under the baseline scenario; *NINF_AS1i* and *NINF_AS2i* represent the annual number of infected dogs imported into mainland France under alternative Scenarios #1 and #2, respectively, and under a specific compliance level (*i*); and *NIMP* is the total number of dogs imported annually into mainland France for commercial purposes.

### 2.7 BLCA, QRA, and Sensitivity Analysis Implementation

The BLCA, QRA model and sensitivity analysis were implemented in R (version 4.4.1) (R Core Team 2024) using R Studio (Posit Team 2024). For the BLCA, the software JAGS software was also employed, along with the “rjags” and “runjags” R packages. The “mc2d” R package (Pouillot and Delignette‐Muller 2010) was used to define the distributions in the QRA model and run simulations (with 100,000 iterations for each simulation). Output convergence was assessed graphically using the “converge” function to ensure that enough simulations were performed. Sensitivity analysis was conducted using the “tornado” function. Results are presented as the mean and median of the posterior distribution for true seroprevalence, along with its 95% probability interval (PI), corresponding to the 2.5th and 97.5th percentiles of the Markov chain Monte Carlo (MCMC) output.

## 3 Results

### 3.1 Estimation of True Seroprevalence of *B. canis* Infection by Country Cluster

**FIGURE 4:**
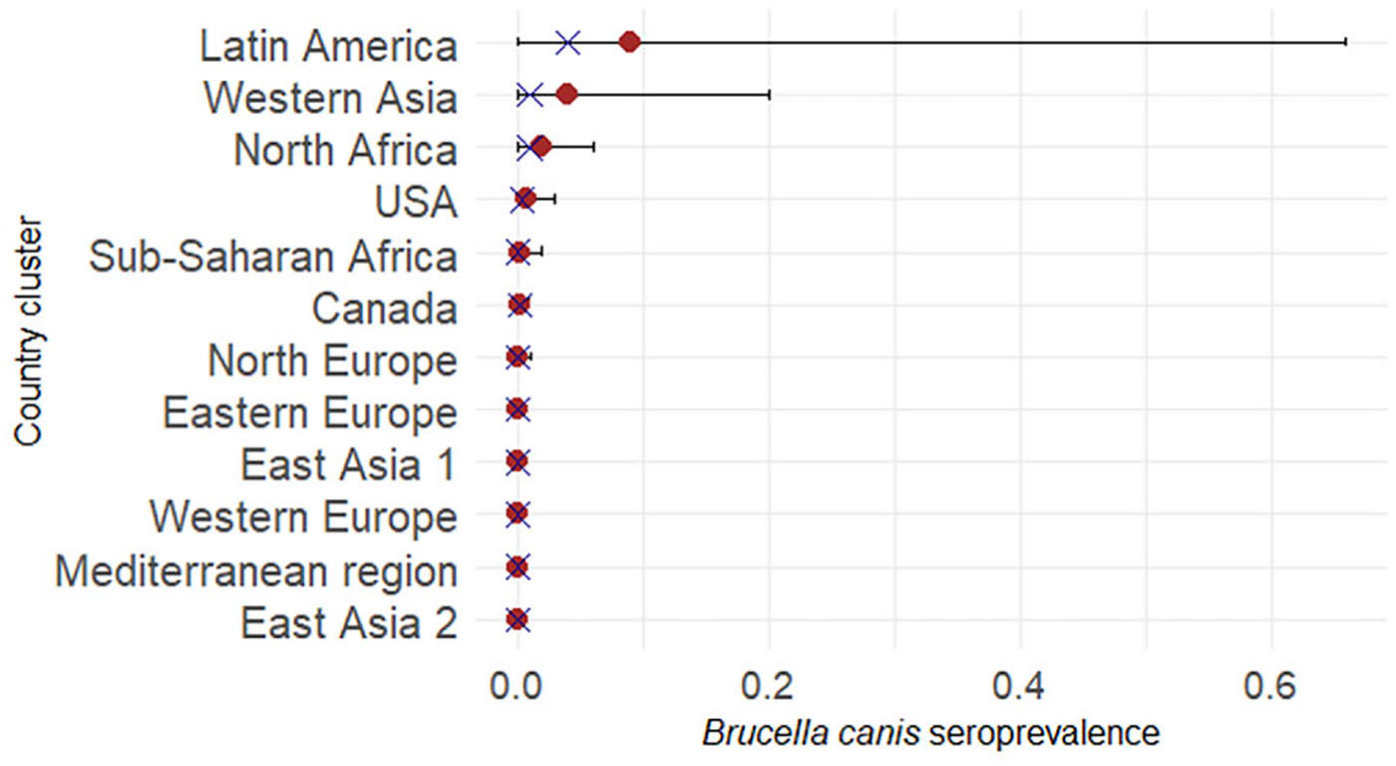
Posterior estimates of medians (crosses) and means (dots) for *Brucella canis* seroprevalence by country cluster along with their 95% probability intervals (bars).

Overall, using BCLA, the posterior estimates of the true seroprevalence of *B. canis* infection by country cluster were low with very wide 95% PI (Figure 4). Four country clusters exhibited higher median posterior estimates: the Latin American cluster (median value = 0.04; 95% PI: 5.1 × 10^−5^–0.66), the Western Asian cluster (median value = 0.01; 95% PI: 1 × 10^−5^–0.20), the North African cluster (median value = 0.01; 95% PI: 0.0003–0.06) and the USA (median value = 0.004; 95% PI: 0.0002–0.03).

### 3.2 Risk Assessment

**FIGURE 5:**
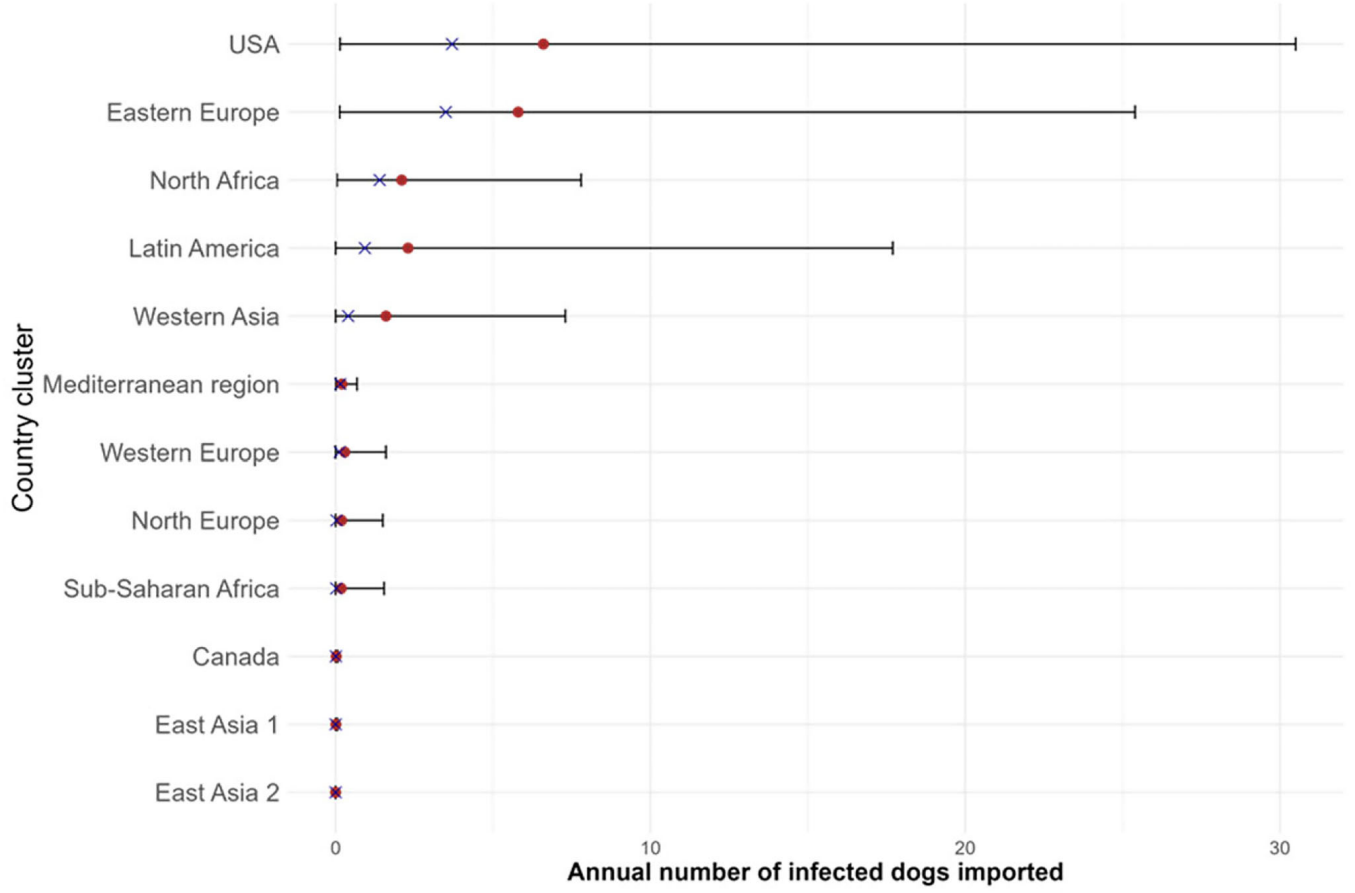
Estimates of the median (crosses) and mean (dots) of annual number of *Brucella canis* infected dogs imported into mainland France by country cluster along with their 95% probability intervals (bars).

Figure 5 shows the results of the risk assessment by country cluster. Four country clusters provided a high annual number of *B. canis* infected dog introductions into mainland France: the USA (median value = 3.70; 95% PI: 0.14–30.50), the Eastern European cluster (median value = 3.50; 95% PI: 0.13–25.40), the North African cluster (median value = 1.40; 95% PI: 0.05–7.80), and the Latin American cluster (median value = 0.93; 95% PI: 0.001–17.70). The global risk (*NINF*) was high with a median value of 41.60 (95% PI: 5.12–8784.00).

### 3.3 Sensitivity Analysis

**FIGURE 6:**
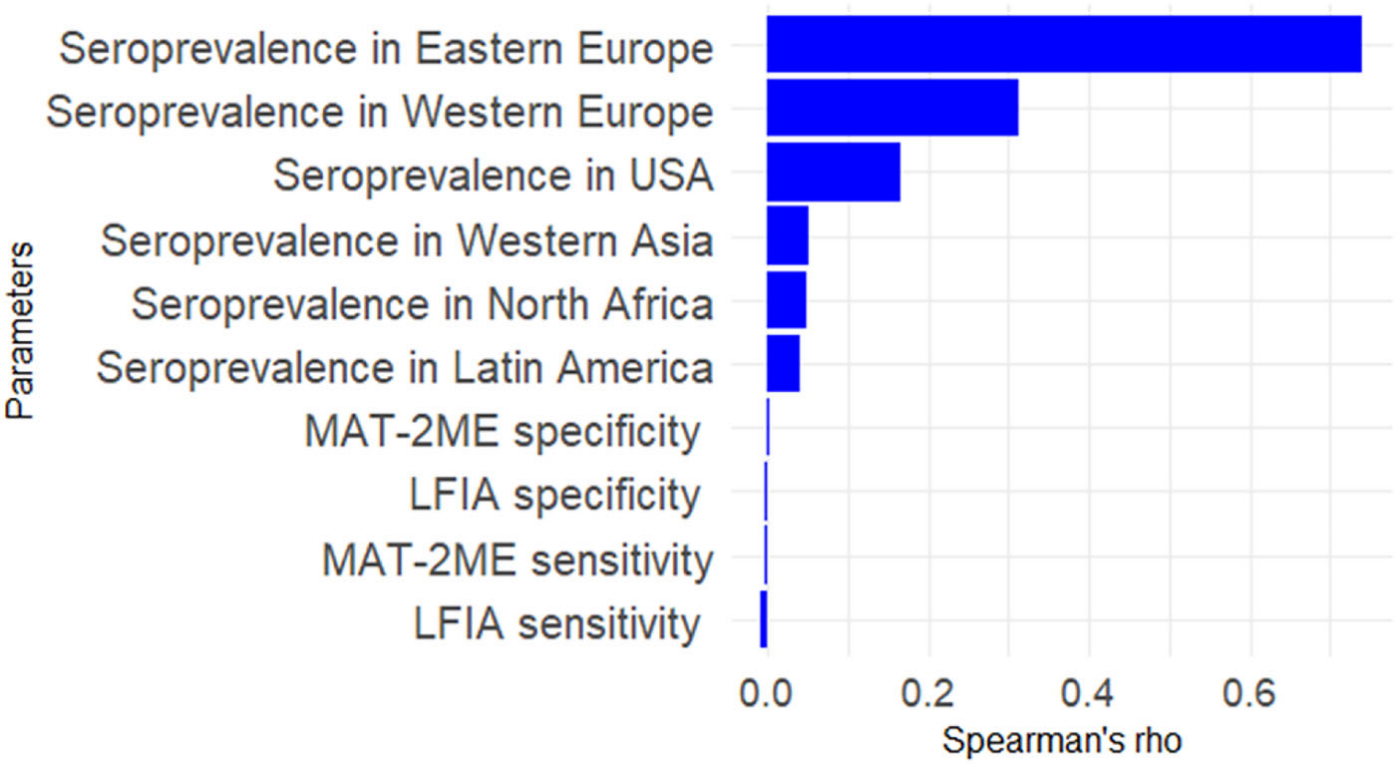
Results of the sensitivity analysis based on Spearman's correlation.

Sensitivity analysis using Spearman's correlation indicated that uncertainty in seroprevalence by country cluster (Eastern and Western Europe, USA, Western Asia, North Africa, and Latin America) had the greatest impact on the uncertainty of the annual number of *B. canis* infected dogs introduced into mainland France (*NINF*) from worldwide, with a strong positive correlation (Figure 6). The sensitivities of MAT‐2ME and LFIA and specificity of MAT‐2ME showed weakly positive correlations, while LFIA specificity exhibited a weak negative correlation with the output parameter.

### 3.4 Alternative scenario Assessed

#### 3.4.1 Scenario #1: Mandatory Pre‐Import Testing

**FIGURE 7:**
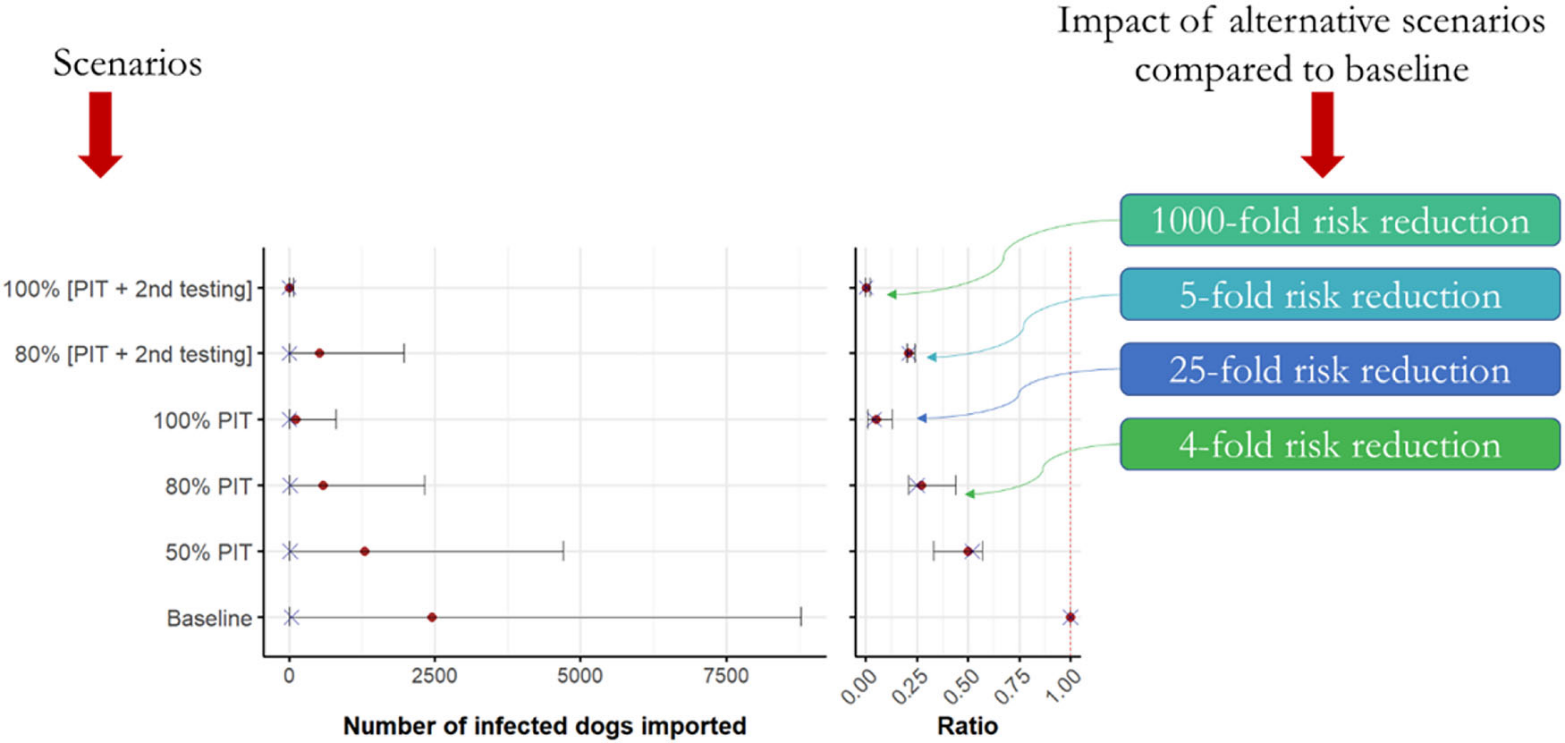
Impacts of the management measures (alternative scenario) on the median (crosses) and mean (dots) of annual number of *Brucella canis* infected dogs imported into mainland France. *PIT*: pre‐import testing for *B. canis*. Ratio represent the number of infected dogs imported into mainland France under the evaluated alternative scenario divided by the number of infected dogs imported under the baseline model.

The introduction of mandatory pre‐import testing for *B. canis* significantly reduced the median annual number of *B. canis*‐infected dogs imported into mainland France. At 50% compliance, the intervention reduced this number twofold, with a median RR of 0.52 (95% PI: 0.33–0.57) compared with baseline. At 80% compliance, the intervention reduced this number fourfold, with a median RR of 0.25 (95% PI: 0.21–0.31) compared with baseline. At 100% compliance, the reduction was 25‐fold, with a median RR of 0.04 (95% PI: 0.005–0.13) compared with baseline (Figure 7).

#### 3.4.2 Scenario #2: Combination of Mandatory Pre‐Import Testing and Additional Testing at the French Border

The introduction of mandatory pre‐import testing combined with additional testing at the French border significantly reduced the median annual number of *B. canis*‐infected dogs imported into mainland France. At 80% compliance, the intervention reduced this number approximately fivefold, with a median RR of 0.21 (95% PI: 0.20–0.24) compared with baseline. At 100% compliance, the reduction was more than 1000‐fold, with a median RR of 0.001 (95% PI: 0.00002–0.018) compared with baseline (Figure 7).

The *ARR*, expressed as the number of *B. canis*‐infected dogs prevented per 1000 imports, varied across scenarios and compliance levels (Table 3).

**TABLE 3:** Absolute risk reduction per 1000 imported dogs under each scenario and compliance level.

Scenario	Compliance	Median ARR (per 1000 imports)	Mean ARR (per 1000 imports)	95% Probability interval
Pre‐import testing (AS1)	50%	1.88	95.6	[0.207–349]
Pre‐import testing (AS1)	80%	2.43	154	[0.309–563]
Pre‐import testing (AS1)	100%	3.27	193	[0.403–705]
Pre‐import + border testing (AS2)	80%	2.70	159	[0.333–574]
Pre‐import + border testing (AS2)	100%	3.42	202	[0.421–723]

For the pre‐import testing scenario (AS1), the median *ARR* increased from 1.88 cases prevented per 1000 imports with 50% compliance to 3.27 cases with 100% compliance.

For the combined pre‐import and border testing scenario (AS2), the *ARR* ranged from 2.70 to 3.42 infected dogs prevented per 1000 imports at 80% and 100% compliance, respectively. The 95% PIs for these estimates remained wide in all scenarios, reflecting uncertainties in the underlying input parameters (Table 3).

## 4 Discussion

This study presented the first QRA addressing the risk of *B. canis* introduction into mainland France through the commercial dog imports. The findings indicate that the probability of introduction of *B. canis* through the commercial dog imports into mainland France is very high, particularly from specific country clusters such as the USA, Eastern Europe, North Africa, and Latin America.

The risk estimates generated by our model reflect the estimated prevalence within each cluster and the declared number of imported dogs. The USA appears to be one of the main sources of *B. canis* in dogs imported for commercial purposes into mainland France, with an estimated median of four infected dogs introduced annually. This estimate reflects the relatively high volume of dog imports and the moderate prevalence levels reported in available studies (Daly et al. 2020; Hubbard et al. 2018; Whitten et al. 2019). Approximately 900 dogs were commercially imported during the study period, making it the third‐largest source of such imports among the twelve clusters considered. Previous investigations conducted over the past two decades, have reported *B. canis* seroprevalence ranging from 0.1% to 5.0% in dogs from animal shelters (Daly et al. 2020; Hubbard et al. 2018; Whitten et al. 2019), especially in southern states (Carmichael 2012; Carmichael and Joubert 1988). Carmichael and Joubert (1988) reported that canine brucellosis was most commonly found in large kennels in the USA, particularly those lacking proper control measures. These reported seroprevalence values are consistent with the posterior distribution obtained for the USA in our BLCA, which yielded a median posterior true seroprevalence of 0.4% and a 95% PI ranging from 0.02% to 3.0%. The risk signal attributed to the USA should be interpreted as the combined effect of significant import volumes and moderate infection rates in certain subpopulations, which did not include high‐standard breeding facilities.

As for the Eastern Europe cluster, although the posterior *B. canis* true seroprevalence was low, it represented the second largest source of infected dogs in the risk assessment, with an estimated median of approximately four infected dogs introduced annually. This outcome is probably due to the high number of imported dogs from this region. Data on dog imports from Eastern Europe to France indicate an increasing trend since 2020, which may have contributed to the rise in detected cases. Remarkably, before 2020, only two cases of *B. canis* infection were reported. However, between 2020 and 2022, 26 infected dogs were identified in France, most of which were imported from Russia, Belarus, and Romania through online purchases (Haut Conseil de la Santé Publique 2022). If this import trend persists, the future introduction of infected dogs from Eastern Europe into France is likely to increase. Other studies have also identified Eastern Europe as the primary source of *B. canis*‐infected dogs in several European countries, including the Netherlands, Germany, Sweden (Buhmann et al. 2019; Dijk et al. 2021; Holst et al. 2012), the United Kingdom (Escauriaza et al. 2021; Süer et al. 2023), as well as Belgium, Italy, Austria, Switzerland, Finland, Denmark, and Norway (Buhmann et al. 2019).

Regarding the Latin American cluster, the study indicates that the *B. canis* true seroprevalence within this cluster was high. This likely explains its ranking as one of the top four sources of the pathogen for mainland France, with an estimated median of one infected dog potentially imported annually. A distinctive feature of this cluster is the abundance of available seroprevalence surveys, which were used to estimate the true posterior seroprevalence (Agudelo‐Flórez et al. 2012; de Aguiar et al. 2005; Almeida et al. 2004; Azevedo et al. 2004; Castrillón‐Salazar et al. 2013; Cavalcanti et al. 2006; Ferreira et al. 2007; Galarce et al. 2020; Giraldo Echeverri et al. 2009; Moraes et al. 2002; Porto et al. 2008; Silva et al. 2012; Tuemmers et al. 2013). This contributes to more precise results within this cluster and helps explain why it is among the primary sources of *B. canis* for France. Furthermore, several of these studies highlight the absence of canine brucellosis control and prevention regulations in the region, a factor that can contribute significantly to the risk of international spread of *B. canis*.

The results also showed that the risk of introducing *B. canis* through the importation of dogs from other clusters of Europe Union countries (Western Europe, Mediterranean region, North Europe) was very low but not null. Residual risk exists, that could be increased by less stringent sanitary controls for dogs imported from these countries since they belong to the EU. In addition, the lack of systematic checks on dogs entering from North Africa, especially through some Mediterranean EU entry points, could facilitate the introduction of *B. canis*.

Several management measures aimed at reducing the risk of *B. canis* introduction were evaluated. Our findings suggest that mandatory pre‐import testing for *B. canis*, with 50%, 80% compliance or full enforcement, could significantly reduce the risk of its introduction into mainland France. In this scenario, the intervention was estimated to prevent a median of 1.88–3.27 cases of *B. canis* infection per 1000 imported dogs, corresponding to compliance levels ranging from 50% to 100%. While this aligns with recommendations proposed by organizations and experts to prevent *B. canis* infections (Boyden 2022, 2024; Bramlage et al. 2015; Buhmann et al. 2019; Cosford 2018; Haut Conseil de la Santé Publique 2022; Hensel et al. 2018; Hollett 2006; Vets 2021), our study is the first to quantitatively evaluate the effectiveness of this strategy. In addition to playing a critical role in preventing the introduction of *B. canis*, mandatory pre‐import testing offers additional benefits. As there is no reliable, effective treatment to cure *B. canis* infections in dogs, and relapse is common after therapy, early detection is critical. It helps prevent further transmission and supports timely decisions about case management. These decisions include isolation, reproductive restrictions, and euthanasia when necessary. Countries that have implemented strict dog import regulations, such as mandatory pre‐import testing (e.g., Australia and New Zealand), remain free of *B. canis*, demonstrating the potential benefits of such preventive measures (Buhmann et al. 2019; Hensel et al. 2018; Medveczky and Crichton 1986).

However, the success of this management strategy depends heavily on strict compliance and robust enforcement. Sub‐optimal compliance or fraudulent certification could significantly undermine its effectiveness. In addition, the illegal trade of dogs outside of any regulatory oversight represents a significant and difficult‐to‐quantify risk because it can bypass official controls entirely. Strict compliance and effective enforcement require additional efforts to overcome associated challenges. Key actions include providing border personnel with comprehensive training, improving traceability systems, and strengthening international collaboration on import controls. These efforts are essential for properly implementing, detecting, and monitoring legal and illegal dog movements. There is also an urgent need to enhance global surveillance and establish harmonized diagnostic protocols for *B. canis* infection. This need arises from the lack of specific clinical manifestations in infected dogs and variability in testing procedures and result interpretation across countries (Baldi et al. 1997; Carmichael et al. 1984; Cosford 2018; Djokic et al. 2023; Keid et al. 2007, 2017; Kimura et al. 2008; Mateu‐de‐Antonio and Martín 1995; Mateu‐de‐Antonio et al. 1993, 1994; Mol et al. 2020; Perletta et al. 2023; Santos et al. 2021; Wanke 2004; Zoha and Carmichael 1982). Furthermore, unlike other *Brucella* species, *B. canis* is not currently classified as a notifiable disease by the World Organization for Animal Health (WOAH) or the World Health Organization (WHO). In France, although canine brucellosis is classified as a regulated and, therefore notifiable disease, specific surveillance and management measures have yet to be implemented (Haut Conseil de la Santé Publique 2022). This lack of a dedicated surveillance system for *B. canis* infection in dogs increases the likelihood that it will remain an underreported pathogen, posing an ongoing risk to both canine and human populations. In the context of free trade policies, a harmonized approach at the EU level would be more appropriate than measures implemented solely at the national (French) level. Therefore, a more realistic and effective way to mitigate the introduction of *B. canis* through intra‐European trade would be to implement risk‐based strategies and harmonized EU‐level regulations.

A more robust prevention strategy combining mandatory pre‐import testing and additional testing at the French border appears to offer enhanced protection. This approach benefits from a second test at the border, which serves as a crucial safeguard against undetected infections. This is particularly important in cases where pre‐import testing alone may fail to detect infected animals due to latent infections, incubation periods, recent exposure, infections occurring after testing, false negatives, or technical errors. Secondary testing increases the likelihood of identifying seroconversion that may occur between the initial screening and the dog's arrival in France, which significantly reduces the risk of introducing *B. canis* into the country. In addition, a significant limitation of serological testing for *B. canis* is its reduced sensitivity during the initial stages of infection when antibody levels may be undetectable. Consequently, dogs infected shortly before sampling may yield false‐negative results due to the diagnostic window period. Furthermore, if testing is conducted well in advance of importation, there is a risk of exposure after testing but before departure, particularly in the absence of quarantine or other controlled management measures. These risks are not fully accounted for by the test's analytical sensitivity alone, and they were not explicitly modeled in the present work. Although we did not explicitly model time since infection or include a dedicated quarantine node, the structure of alternative Scenario #2 implicitly assumes that dogs are temporarily held at the border until post‐entry test results are available. This setup mirrors post‐entry quarantine protocols implemented in countries such as Australia, where imported animals are housed under controlled conditions pending health clearance. However, this approach may present additional challenges, including increased implementation complexity and cost. To support its adoption, a cost–benefit analysis should be conducted to assess its feasibility and justify its potential benefits.

Another key consideration is serological screening in low‐prevalence contexts, which carries a significant risk of false‐positive results, especially when multiple testing steps are involved. This trade‐off is well recognized in surveillance and prevention programs targeting rare but high‐impact diseases. The resulting low positive predictive value can lead to avoidable costs and psychological stress for owners. In extreme cases, it can result in inappropriate treatment or even euthanasia. Nevertheless, the potential consequences of importing even a single infected dog, especially in the canine breeding sector, warrant preventive measures. These consequences include reproductive failure, persistent infection within kennels, loss of valuable genetic lines, economic losses for breeders, and the risk of zoonotic transmission. From a One Health perspective, the long‐term harm associated with failing to prevent the introduction of an infection may outweigh the short‐term challenges of managing a limited number of false positives. Ultimately, we agree that a dedicated benefit–risk analysis would be highly relevant for future work. Such an analysis should consider test performance, prevalence, the economic and ethical implications of false positives, the program's overall cost‐effectiveness, and the threshold at which preventive actions are justified. This would support more refined, evidence‐based decision‐making regarding *B. canis* surveillance.

The findings of our study must be interpreted with caution, as they rely on certain assumptions due to limited consistent data availability on *B. canis* seroprevalence. Specifically, identical seroprevalence of *B. canis* infection within each cluster was assumed, although regional variations likely exist. Most clusters were geographically large, often spanning entire continents, and the available samples were not always representative of the study population. Efforts were made to restrict import data to dogs imported for commercial purposes. However, it was not possible to apply the same restriction to seroprevalence data because very few studies have specifically estimated the prevalence of *B. canis* in breeding kennels. In many countries, available data comes from shelters, pets, strays, or broader, often undefined, canine populations. Consequently, the seroprevalence estimates used in the model were not always representative of breeding dogs. This limitation underscores the importance of interpreting model outputs with caution and emphasizes the necessity of conducting targeted seroprevalence studies in breeding dog populations.

In addition, previously published data on apparent seroprevalence were obtained in situations where none of the tests used was a perfectly accurate gold standard. To address this limitation, a BLCA was applied, allowing to account for uncertainty in seroprevalence data and to obtain estimates of true seroprevalence by cluster despite the use of imperfect diagnostic tests. Despite the problem of representativeness in the available seroprevalence studies which cannot be fixed through statistical methods, BLCA was a useful component of the QRA because of its ability to integrate different data sources by estimating true prevalence values, even when the data sources themselves were heterogeneous. This method enhanced the robustness and accuracy of the model by accounting for uncertainties and the limitations of imperfect diagnostic tests. Future studies should prioritize the generation of more reliable and representative seroprevalence data, especially from high‐risk regions, as this would greatly improve risk estimates.

## 5 Conclusion

The significant reproductive disorders caused by *B. canis* infections in dogs, particularly those used for commercial breeding, along with other significant clinical signs, such as discospondylitis, uveitis, and lymphadenopathy, and its zoonotic potential, underscore the urgency of implementing management measures to prevent the introduction of the pathogen into mainland France. This study contributes to the understanding of the role commercial dog imports play in the introduction of the pathogen into mainland France. In addition, it provides a flexible model that can be readily updated, as new data become available. This could allow animal and public health managers to generate up‐to‐date results that could guide the implementation of *B. canis* surveillance and control measures. In addition, the model serves as a decision‐making tool to reduce the risk of *B. canis* introduction and spread.

## Supporting information




**Supporting Appendix A.1**: Published data on apparent seroprevalence of *Brucella canis* infection by cluster of countries used in the Bayesian approaches. **Supporting Appendix A.2**: Published data on *Brucella canis* seropositivity proportions by European country clusters used in Bayesian analyses. **Supporting Appendix B**: Published sensitivity data for *Brucella canis* serological tests: minimum, maximum and 5^th^ percentile values for prior beta distribution parameter estimation. **Supporting Appendix C**: Published specificity data for *Brucella canis* serological tests: minimum, maximum and 5^th^ percentile values for prior beta distribution parameter estimation. **Supporting Appendix D**: Mean annual numbers of dogs imported into France for commercial purposes from worldwide sources over the period 2021 ‐2023 by cluster of countries (Ingenium animalis, 2024)
