# Supplementary material for: Risk Assessment and Management of Brucella canis Introduction via Commercial Dog Imports Into France
Source: Risk Anal. 2026 Mar 14;46(4):e70217. doi: 10.1111/risa.70217 (PMC12988457; doi:10.1111/risa.70217)
Supplement: Supplementary file 1 — Supporting Appendix A.1: Published data on apparent seroprevalence of Brucella canis infection by cluster of countries used in the Bayesian approaches. Supporting Appendix A.2: Published data on Brucella canis seropositivity proportions by European country clusters used in Bayesian analyses. Supporting Appendix B: Published sensitivity data for Brucella canis serological tests: minimum, maximum and 5th percentile values for prior beta distribution parameter estimation. Supporting Appendix C: Published specificity data for Brucella canis serological tests: minimum, maximum and 5th percentile values for prior beta distribution parameter estimation. Supporting Appendix D: Mean annual numbers of dogs imported into France for commercial purposes from worldwide sources over the period 2021 ‐2023 by cluster of countries (Ingenium animalis, 2024) [file RISA-46-0-s001.docx]

**Supplemental material - Risk assessment and management of *Brucella canis* introduction via commercial dog imports into France**

Appendix A.1. Published data on apparent seroprevalence of *Brucella canis* infection by cluster of countries used in the Bayesian approaches

| **Location** | **Sample Population** | **Prevalence (Number of positive samples/total number of samples)** | **Test** | **Year** | **Reference** |
| --- | --- | --- | --- | --- | --- |
| **USA** | | | | | |
| **Mississippi, USA** | Shelter | 2.28 (13/571) | RSAT, 2ME-RSAT | 2018 | Hubbard et al., 2018 |
| **Minnesota, USA** | Shelter | 0.1 (1/943) | RSAT, 2ME-RSAT | 2019 | Whitten et al., 2019 |
| **South Dakota, USA** | Shelter | 5.1 (45/889) | RSAT, 2ME-RSAT | 2019 | Daly et al., 2020 |
|  |  |  |  |  |  |
| **CANADA** | | | | | |
| **Ontario, Canada** | Breeding kennel | 11.76 (127/1080) | RSAT, 2ME-RSAT | 2020 | Weese, 2020 |

RSAT: Rapid slide agglutination test

2ME-RSAT: 2-mercaptoethanol rapid slide agglutination test

| **Location** | **Sample Population** | | **Prevalence**  **(Number of positive samples/Total number of samples)** | | **Test** | **Year** | **Reference** |
| --- | --- | --- | --- | --- | --- | --- | --- |
| **LATIN AMERICA** | | | | | | | |
| **Botucata, Sao Paulo State, Brazil** | | Pet | | 0.84 (9/1073) | RSAT, 2ME-RSAT | 2002 | Moraes et al., 2002 |
| **Santana de Parnaiba, Sao Paulo, Brazil** | | Pet | | 3.65 (15/410) | AGID, 2ME-AGID | 2003-2004 | Azevedo et al., 2004 |
| **Alfensa, Minas Gerais, Brazil** | | Pet | | 14.20 (90/635) | AGID | 2004 | Almeida et al., 2004 |
| **Salvador, Brazil** | | Pet | | 5.88 (5/85) | AGID | 2006 | Cavalcanti et al., 2006 |
| **Callao, Peru** | | Breeding kennel | | 15.57 (71/456) | AGID | 2006 | Ramírez et al., 2012 |
| **Rio de Janeiro, Brazil** | | Pet | | 2.53 (8/316) | AGID | 2007 | Ferreira et al., 2007 |
| **Campina Grande, Paraiba, Brazil** | | Random | | 2.35 (4/170) | AGID | 2008 | Vasconcelos et al., 2008 |
| **Maceio-Alagoas, Brazil** | | Pet | | 3.30 (3/90) | AGID, 2ME-AGID | 2008 | Porto et al., 2008 |
| **Buenos Aires, Argentina** | | Mixed | | 7.30 (16/219) | RSAT | 2008 | Boeri et al., 2008 |
| **Buenos Aires Province, Argentina** | | Shelter | | 10.70 (24/227) | RSAT,  ELISA | 2009 | Lopez et al., 2009 |
| **Antioquia, Colombia** | | Breeding kennel | | 11.31 (166/1467) | 2ME-RSAT | 2009 | Giraldo-Echeverri et al., 2009 |
| **Medellin, Colombia** | | Stray | | 6.78 (15/221) | 2ME-RSAT | 2010 | Ruiz et al., 2010 |
| **Araguaina, Tocantins, Brazil** | | Pet | | 44.53  (167/374) | AGID | 2011 | Dorneles et al., 2011 |
| **Paraiba state, Brazil** | | Pet | | 3.11 (6/193) | AGID | 2011 | Fernandes et al., 2011 |
| **Apucarana & Londrina, Brazil** | | Shelter | | 4.00 (4/100) | AGID | 2012 | Silva et al., 2012 |
| **Medellin, Colombia** | | Pet | | 2.76 (12/441) | ICT | 2012 | Agudelo-Flórez et al., 2012 |
| **Ilheus, Brazil** | | Pet and stray | | 3.45 (22/638) | AGID | 2012 | Bezerra et al., 2012 |
| **Parana, Brazil** | | Shelter | | 2.85 (5/175) | AGID | 2013 | de Paula Dreer et al., 2013 |
| **Rio Grande do Norte state, Brazil** | | Pet | | 28.90 (120/416) | AGID | 2013 | Fernandes et al., 2013 |
| **Araguaina, Tocantins, Brazil** | | Pet | | 54.77 (132/241) | AGID | 2013 | Santana et al., 2013 |
| **Antioquia, Colombia** | | Breeding kennel | | 14.98 (64/428) | 2ME-RSAT | 2013 | Castrillón-Salazar et al., 2013 |
| **Temuco city, Chile** | | Shelter | | 1.00 (4/400) | ICT | 2013 | Tuemmers et al., 2013 |
| **Curicó city, Chile** | | Breeding kennel | | 18.18 (6/33) | ICT | 2013 | Troncoso et al., 2013 |
| **Lima, Peru** | | Pet | | 4.86 (14/288) | AGID | 2016 | Maza & Morales, 2016 |
| **Concepción, Paraguay** | | Pet | | 9.62 (5/52) | ICT | 2017 | Colman et al., 2017 |
| **Bogota, Colombia** | | Shelter | | 1.96 (1/51) | LFIA | 2021 | Laverde et al., 2021 |
| **Central Valley, Costa Rica** | | Mixed (breeding kennel, pet) | | 6.29 (19/302) | AGID | 2021 | Suárez-Esquivel et al., 2021 |
| **Curitiba, Brazil** | | Pet | | 4.54 (12/264) | 2ME-MAT, LFIA | 2024 | Schiavo et al., 2024 |

RSAT: Rapid slide agglutination test

2ME-RSAT: 2-mercaptoethanol rapid slide agglutination test

AGID: Agar Gel Immunodiffusion test

2ME-AGID: 2-mercaptoethanol Agar Gel Immunodiffusion test

ELISA: Enzyme-Linked Immunosorbent Assay

ICT: Immunochromatographic Test

LFIA: Lateral Flow Immunochromatographic assay test

2ME-MAT: 2-mercaptoethanol Microscopic Agglutination Test

| **Location** | **Sample Population** | **Prevalence**  **(Number of positive samples/Total number of samples)** | **Test** | **Year** | **Reference** |
| --- | --- | --- | --- | --- | --- |
| **EAST ASIA 1** | | | | | |
| **India** | Pet | 4.30  (34/787) | ELISA | 2008 | Mathur et al., 2008 |
| **Beijing, China** | Breeding kennel | 48.75 (39/80) | RSAT | 2012 | Jiang et al., 2012 |
| **Beijing, China** | Mixed (breeding kennel, pet) | 1.33 (16/1200) | TAT | 2013 | Xiang et al., 2013 |
| **Rajasthan, India** | Stray | 6.75 (16/237) | ELISA | 2014 | Yoak et al., 2014 |
|  |  |  |  |  |  |
| **EAST ASIA 2** | | | | | |
| **Kanawaga, Japan** | Pet | 2.47 (12/485) | MAT | 2008 | Kimura et al., 2008 |
| **Tokushima Prefecture, Japan** | Mixed (breeding, kennel, pet) | 2.47 (12/485) | MAT | 2013 | Kume et al., 2013 |

ELISA: Enzyme-Linked Immunosorbent Assay

RSAT: Rapid Slide Agglutination Test

TAT: Tube Agglutination Test

MAT: Microscopic Agglutination Test

| **Location** | **Sample Population** | | **Prevalence**  **(Number of positive samples/Total number of samples)** | **Test** | **Year** | **Reference** |
| --- | --- | --- | --- | --- | --- | --- |
| **WESTERN ASIA** | | | | | | |
| **Istanbul & Izmir, Turkey** | | Shelter | 7.45 (27/362) | ELISA | 2005 | Oncel & Akan, 2005 |
| **Istanbul & Izmir, Turkey** | | Shelter | 7.73 (28/362) | TAT, 2ME-TAT | 2005 | Oncel & Akan, 2005 |
| **Ahvaz, Iran** | | Pet | 4.90 (5/102) | ICT | 2009 | Mosallanejad et al., 2009 |
| **Aydın, İzmir & Muğla, Turkey** | | Mixed (Shelter, Pet) | 6.00 (12/200) | 2ME-RSAT | 2019 | Parin et al. 2020 |
| **Amman, Jarash, Irbid, Ajloun, Al-Shouneh, Al-Ramtha & Jawa,  Jordan** | | Mixed (Police, Breeding, Shelter) | 8.28 (14/169) | RSAT, 2ME-RSAT | 2019 | Alshehabat et al., 2019 |
| **Manisa, Turkey** | | Shelter | 37.14 (13/35) | RSAT | 2023 | Özavci et al., 2023 |

ELISA: Enzyme-Linked Immunosorbent Assay

TAT: Tube Agglutination Test

2ME-TAT: 2-mercaptoethanol Tube Agglutination Test

ICT: Immunochromatographic Test

RSAT: Rapid Slide Agglutination Test

2ME-RSAT: 2-mercaptoethanol Rapid Slide Agglutination Test

| **Location** | **Sample Population** | **Prevalence**  **(# Positive samples/Total samples)** | **Test** | **Year** | **Reference** |  |
| --- | --- | --- | --- | --- | --- | --- |
| **NORTH AFRICA** | | | | | |  |
| **Greater Cairo region & Damietta governorate,  Egypt** | Mixed (Stray, Pet) | 3.79 (17/449) | RSAT, 2ME-RSAT | 2023 | Hamdy et al., 2023 |  |
|  |  |  |  |  |  |  |
| **SUB-SAHARAN AFRICA** | | | | | |  |
| **Lagos State, Nigeria** | Pet | 0.27 (1/366) | RSAT | 2011 | Cadmus et al., 2011 |  |
| **Harare, Zimbabwe** | Pet | 17.60 (57/324) | ELISA | 2013 | Chinyoka et al., 2014 |  |
|  |  |  |  |  |  |  |
| **Eastern Cape, South Africa** | Mixed (Stray, Shelter) | 9.75 (39/400) | TAT, 2ME-TAT | 2017 | Charné Etsebeth, 2017 |  |
| **Gauteng, the Eastern Cape & Western Cape provinces, South Africa.** | Mixed (Breeding, Shelter) | 4.36 (52/1191) | 2ME-RSAT | 2019 | Oosthuizen et al., 2019 |  |
| **Enugu & Anambra States, Nigeria** | Mixed (Slaughter, Stray, Pet) | 27.64 (34/123) | ELISA | 2020 | Anyaoha et al., 2020 |  |

RSAT: Rapid Slide Agglutination Test

2ME-RSAT: 2-mercaptoethanol Rapid Slide Agglutination Test

ELISA: Enzyme-Linked Immunosorbent Assay

TAT: Tube Agglutination Test

2ME-TAT: 2-mercaptoethanol Tube Agglutination Test

Appendix A.2. Published data on Brucella canis seropositivity proportions by European country clusters used in Bayesian analyses

| **Country cluster** | **Countries** | **Test** | **Minimum - Maximum seropositivity proportions** | **References** |
| --- | --- | --- | --- | --- |
| Western Europe | Austria, Belgium, Germany, Luxembourg, Monaco, Netherlands, Switzerland | Tube Agglutination Test (TAT) | 0.00 – 0.12 | Buhmann et al., 2019 |
| Eastern Europe | Albania, Belarus, Bulgaria, Czech Republic, Croatia, Hungary, Moldavia, Poland, Romania, Russia, Slovakia, Slovenia, Ukraine | TAT | 0.00 – 0.04 | Buhmann et al., 2019 |
| Northern Europe | Denmark, Estonia, Finland, Island, Ireland, Latvia, Lithuania, Norway, United Kingdom, Sweden | TAT | 0.00 – 0.14 | Buhmann et al., 2019 |
| Mediterranean Region of Europe | Andorra, Vatican City, Greece, Gibraltar, Italy, Malta, Portugal, Spain | TAT | 0.00 – 0.08 | Buhmann et al., 2019 |

Appendix B. Published sensitivity data for *Brucella canis* serological tests: minimum, maximum and 5^th^ percentile values for prior Beta distribution parameter estimation

| **Serological test** | **Minimum -Maximum** | **Prior**  **most likely value of sensitivity** | **References** | | **5% percentile** | **Beta prior distribution parameters used for sensitivity** | |
| --- | --- | --- | --- | --- | --- | --- | --- |
|  |  |  |  |  |  | **a** | **b** |
| RSAT | [0.60 – 1.00] | 0.71 | Hollett, 2006; Keid et al., 2009; Cosford, 2018 | 0.60 | | 40.131 | 16.983 |
| 2ME-RSAT | [0.00 – 1.00] | 0.32 | Cosford, 2018; Djokic et al., 2023 | 0.70 | | 2.324 | 3.813 |
| TAT | [NA – 1.00] | 0.71 | Similarity assumption with RSAT - Hollett 2006; Cosford, 2018; Djokic et al., 2023 | 0.60 | | 40.131 | 16.983 |
| 2ME-TAT | [0.00 – 1.00] | 0.32 | Similarity assumption with RSAT-2ME - Cosford, 2018; Djokic et al., 2023 | 0.70 | | 2.324 | 3.813 |
| AGID (*B. canis*) | [0.61 – 0.96] | 0.87 | Djokic et al., 2023 | 0.60 | | 9.568 | 2.280 |
| 2ME-AGID (*B. canis*) | NA | 0.50 | Assumption - should be less than the Se of AGID (*B. canis*) because adding 2-ME decreases the Se of a test (Keid et al., 2009; Wanke et al., 2012; Keid et al., 2015; Mol et al., 2020) | 0.60 | | 33.383 | 33.383 |
| AGID (LPS *B. ovis*) | [0.26 – 0.53] | 0.53 | Keid et al., 2009; Keid et al., 2015; Djokic et al., 2023 | 0.20 | | 3.000 | 2.774 |
| 2ME-AGID (LPS *B. ovis*) | NA | 0.30 | Assumption - should be less than the Se of AGID (LPS *B. ovis*) because adding 2-ME decreases the Se of a test (Keid et al., 2009; Wanke et al., 2012; Keid et al., 2015; Mol et al., 2020) | 0.20 | | 6.281 | 13.322 |
| MAT | [0.67 – 0.89] | 0.89 | Keid et al., 2009 ; Wanke et al., 2012 ; Keid et al., 2015 ; Mol et al., 2020 | 0.60 | | 8.685 | 1.950 |
| 2ME-MAT | NA | 0.60 | Assumption - should be less than the Se of MAT (*B. canis*) because adding 2-ME decreases the Se of a test (Keid et al., 2009; Wanke et al., 2012; Keid et al., 2015; Mol et al., 2020) | 0.60ₐ | | 33.387 | 33.387 |
| LFIA | [0.17 – 1.00] | 0.96 | Esfandiari & Klingeborn, 2000; Wanke et al., 2012; Keid et al., 2015; Djokic et al., 2023 | 0.80 | | 19.074 | 1.753 |
| ELISA | [0.88 – 1.00] | 0.95 | Cosford, 2018; Djokic et al., 2023 | 0.90 | | 99.698 | 6.698 |

NA: not available

RSAT: Rapid Slide Agglutination Test

2ME-RSAT: 2-mercaptoethanol Rapid Slide Agglutination Test

TAT: Tube Agglutination Test

2ME-TAT: 2-mercaptoethanol Tube Agglutination Test

AGID: Agar Gel Immunodiffusion test

2ME-AGID: 2-mercaptoethanol Agar Gel Immunodiffusion test

MAT: Microscopic Agglutination Test

2ME-MAT: 2-mercaptoethanol Microscopic Agglutination Test

LFIA: Lateral Flow Immunochromatographic assay test

ELISA: Enzyme-Linked Immunosorbent Assay

Appendix C. Published specificity data for *Brucella canis* serological tests: minimum, maximum and 5^th^ percentile values for prior Beta distribution parameter estimation

| **Serological test** | **Minimum -Maximum** | **Prior**  **most likely value of specificity** | **References** | **5% percentile** | **Beta prior distribution parameters used for specificity** | |
| --- | --- | --- | --- | --- | --- | --- |
|  |  |  |  |  | **a** | **b** |
| RSAT | [0.00 – 1.00] | 0.83 | Keid et al., 2009; Cosford, 2018 | 0.70 | 30.462 | 7.034 |
| 2ME-RSAT | [0.42 – 1.00] | 1.00 | Cosford, 2018; Djokic et al., 2023 | 0.90 | 28.433 | 1.000 |
| TAT | [NA – 1.00] | 0.83 | Similarity assumption with RSAT - Hollett 2006; Cosford, 2018; Djokic et al., 2023 | 0.70 | 30.462 | 7.034 |
| 2ME-TAT | [0.42 – 1.00] | 1.00 | Similarity assumption with RSAT-2ME - Cosford, 2018; Djokic et al., 2023 | 0.90 | 28.433 | 1.000 |
| AGID (*B. canis*) | [0.94 – 1.00] | 0.95 | Djokic et al., 2023 | 0.90 | 99.698 | 6.195 |
| 2ME-AGID (*B. canis*) | NA | 0.99 | Assumption - should be greater than the specificity of AGID (*B. canis*) because adding 2-ME increases the Sp of a test (Keid et al., 2009; Wanke et al., 2012; Keid et al., 2015; Mol et al., 2020) | 0.95 | 88.280 | 1.882 |
| AGID (LPS *B. ovis*) | [n.a – 1.00] | 0.99 | Keid et al., 2008; Djokic et al., 2023 | 0.80 | 14.522 | 1.137 |
| 2ME-AGID (LPS *B. ovis*) | NA | 1.00 | Assumption - should be greater than the Sp of AGID (LPS *B. ovis*) because adding 2-ME increases the Sp of a test (Keid et al., 2009; Wanke et al., 2012; Keid et al., 2015; Mol et al., 2020) | 0.90 | 28.433 | 1.000 |
| MAT | [n.a – 1.00] | 0.97 | Keid et al., 2009; Wanke et al., 2012; Keid et al., 2015; Mol et al., 2020 | 0.90 | 53.581 | 26.260 |
| 2ME-MAT | NA | 0.99 | Assumption - should be greater than the Sp of MAT because adding 2-ME increases the Sp of a test (Keid et al., 2009; Wanke et al., 2012; Keid et al., 2015; Mol et al., 2020) | 0.98 | 100.000 | 2.000 |
| LFIA | [0.94 – 1.00] | 0.99 | Esfandiari & Klingeborn, 2000; Wanke et al., 2012; Keid et al., 2015; Djokic et al., 2023 | 0.80 | 14.522 | 1.137 |
| ELISA | [0.94 – 1.00] | 0.99 | Cosford, 2018; Djokic et al., 2023 | 0.90 | 34.166 | 13.350 |

NA: not available

RSAT: Rapid Slide Agglutination Test

2ME-RSAT: 2-mercaptoethanol Rapid Slide Agglutination Test

TAT: Tube Agglutination Test

2ME-TAT: 2-mercaptoethanol Tube Agglutination Test

AGID: Agar Gel Immunodiffusion test

2ME-AGID: 2-mercaptoethanol Agar Gel Immunodiffusion test

MAT: Microscopic Agglutination Test

2ME-MAT: 2-mercaptoethanol Microscopic Agglutination Test

LFIA: Lateral Flow Immunochromatographic assay test

ELISA: Enzyme-Linked Immunosorbent Assay

Appendix D. Mean annual numbers of dogs imported into France for commercial purposes from worldwide sources over the period 2021 -2023 by cluster of countries (Ingenium animalis, 2024)

| Cluster of countries | Countries | Mean annual number of dogs imported |
| --- | --- | --- |
| Western Europe | Austria, Belgium, Germany, Luxembourg, Monaco, Netherlands, Switzerland | 718 |
| Eastern Europe | Albania, Belarus, Bulgaria, Czech Republic, Croatia, Hungary, Moldavia, Poland, Romania, Russia, Slovakia, Slovenia, Ukraine | 8,137 |
| North Europe | Denmark, Estonia, Finland, Island, Ireland, Latvia, Lithuania, Norway, United Kingdom, Sweden | 159 |
| Mediterranean region of Europe | Andorra, Vatican City, Greece, Gibraltar, Italy, Malta, Portugal, Spain | 2,071 |
| South Asia | Afghanistan, Armenia, Cyprus, United Arab Emirates, Israel, Iraq, Iran, Lebanon, Oman, Pakistan, Qatar, Syria, Turkey | 37 |
| East Asia 1 | Cambodia, China, India, Indonesia, Malaysia, Thailand, Vietnam | 17 |
| East Asia 2 | Japan, Hong-Kong, Republic of Korea, Taiwan | 18 |
| Sub-Saharan Africa | Cameroon, Cape Verde, Gabon, Kenya, Madagascar, Mauritius, Nigeria, Senegal, South African | 82 |
| North Africa | Algeria, Egypt, Morocco, Mauritania, Tunisia | 144 |
| Latin America | Argentina, Bolivia, Brazil, Chili, Ecuador, Mexico, Peru, Porto-Rico, Venezuela | 27 |
| USA | USA | 901 |
| Canada | Canada | 8 |
